# Delineating the field of medical education: Bibliometric research approach(es)

**DOI:** 10.1111/medu.14677

**Published:** 2021-11-02

**Authors:** Lauren A. Maggio, Anton Ninkov, Jason R. Frank, Joseph A. Costello, Anthony R. Artino

**Affiliations:** ^1^ Uniformed Services University of the Health Sciences in Bethesda Maryland USA; ^2^ School of Information Studies University of Ottawa Ottawa Ontario Canada; ^3^ Specialty Education for the Royal College of Physicians and Surgeons Ottawa Ontario Canada; ^4^ Department of Emergency Medicine University of Ottawa Ottawa Ontario Canada; ^5^ Human Function, and Rehabilitation Sciences, Evaluation and Educational Research George Washington University School of Medicine and Health Sciences Washington District of Columbia USA

## Abstract

**Background:**

The field of medical education remains poorly delineated such that there is no broad consensus of articles or journals that comprise ‘the field’. This lack of consensus indicates a missed opportunity for researchers to generate insights about the field that could facilitate conducting bibliometric studies and other research designs (e.g., systematic reviews) and also enable individuals to identify themselves as ‘medical education researchers’. Other fields have utilised bibliometric field delineation, which is the assigning of articles or journals to a certain field in an effort to define that field.

**Process:**

In this *Research Approach*, three bibliometric field delineation approaches—information retrieval, core journals, and journal co‐citation—are introduced. For each approach, the authors describe attempts to apply it in medical education and identify related strengths and weaknesses. Based on co‐citation, the authors propose the Medical Education Journal List 24 (MEJ‐24), as a starting point for delineating medical education and invite the community to collaborate on improving and potentially expanding this list.

**Pearls:**

As a research approach, field delineation is complicated, and there is no clear best way to delineate the field of medical education. However, recent advances in information science provide potentially fruitful approaches to deal with the field's complexity. When considering these approaches, researchers should consider collaborating with bibliometricians. Bibliometric approaches rely on available metadata for articles and journals, which necessitates that researchers examine the metadata prior to analysis to understand its strengths and weaknesses, and to assess how this might affect data interpretation. While using bibliometric approaches for field delineation is valuable, it is important to remember that these techniques are only as good as the research team's interpretation of the data, which suggests that an expanded approach is needed to better delineate medical education, an approach that includes active discussion within the medical education community.

## Abstract 2

How do we draw lines around #MedEd? This article introduces 3 #bibliometric approaches: info retrieval, core journals & journal co‐citation to kickstart the conversation and provide a starter set of journals to build upon.

## 1 BACKGROUND

The field of medical education remains poorly delineated. Over the last decade, multiple researchers have aimed to describe medical education and its outputs using bibliometrics, which is the use of statistics to study books, journal articles, and other publication types.^1^, ^2^, ^3^, ^4^, ^5^, ^6^ To conduct such studies, researchers (including several members of our author team) must make judgement calls about which publications are ‘in’ or ‘out’ of medical education. Researchers who make these calls do so in a fairly ad hoc way because, to our knowledge, there is currently no broad consensus of what constitutes the field of medical education and the articles and journals that comprise it. In this article, we have chosen to refer to medical education as a field based on Beyer and Lodahl's description of a field as ‘providing the structure of knowledge in which faculty members are trained and socialized; carry out tasks of teaching, research, and administration; and produce research and educational output’.^7^ However, we recognise that debate about this distinction exists.^6^, ^8^ This debate, however, is beyond the scope of the present paper. Moreover, to keep the scope of this manuscript manageable, we focus on medical education and not the broader field of health professions education.

The lack of an agreed upon common understanding or delineation—that is, to define or to indicate the position, border or boundary^9^—of the field of medical education signals a missed opportunity to generate insights about the field. These insights would be important not only for conducting bibliometric studies but also for executing other research designs like systematic reviews. For example, the ability to consistently delineate medical education would facilitate setting a basis for citation scores and for an investigator's ability to identify themselves as ‘a medical education researcher’. Thus, in this manuscript, we describe our attempts to use three *field delineation* approaches that rely on bibliometrics: information retrieval, core journals, and journal co‐citation. We recognise that these are only three approaches of many that can be used for field delineation (e.g., consensus building by members of a field), but we believe that these approaches provide a valuable first step. For each approach, we identify strengths and weaknesses and provide practical tips for implementing each approach in medical education. Finally, based on our experiences wrestling with the challenge of field delineation, we invite the medical education community to further collaborate to delineate medical education. To get this conversation started, we introduce a list of 24 journals to serve as a field delineation ‘starter set’ in medical education.

Bibliometric field delineation is described as the assigning of articles or journals to a certain field (i.e., using the field's ‘building blocks’^10^ to define that field^11^). Field delineation itself is often viewed as the first step in a research process to allow scientists to explore the structures and dynamics of a research field using bibliometrics.^12^, ^13^ Bibliometrics is the analysis of published information (e.g., journal articles) and its related metadata (e.g., titles, abstracts) using statistics.^14^ Bibliometrics provides a sense of what is valued, recognised and utilised in a field's scholarly literature.^6^ Several fields, including genomics,^15^ nanoscience^12^ and information science,^11^ have used field delineation to draw boundaries around their fields and then, using these parameters, have described the field's journals, topics, members and trends using bibliometrics. To our knowledge, the field of medical education has not been delineated in this systematic way.

There are practical and psychosocial reasons for delineating the field of medical education. For example, if a researcher wished to update Albert et al's 2007 study in which ‘influential figures’ were interviewed to determine how to prioritise medical education research, they would first need to know the universe of publications within which to begin to identify these individuals.^16^ Similarly, if a researcher wanted to understand if medical education is ‘advancing on big questions’ as Regehr^5^ has implored us to do, then they would need to be aware of what is considered ‘fair game’ for inclusion. Additionally, for a researcher undertaking a systematic review, if they wished to hand search the indices of core journals to supplement their comprehensive database searches, it would be helpful to know to which journals they should dedicate their energy to searching. Without this information, it is difficult to chart our progress and build on our previous successes.

Field delineation also provides the foundation for generating and understanding benchmark metrics about a field. These metrics (e.g., journal impact factor^17^ or H‐index^18^) can be important for a field with researchers who may call a variety of academic departments their home. For example, it would be important for a medical educator to clearly communicate to a chair of Medicine, who is responsible for reviewing promotion packets from a broad variety of researchers, the field delineation benchmarks in medical education, to demonstrate that their research impact aligns with or surpasses others' in their specific field. While use of such benchmarks can be helpful, we advise caution in their application.

In addition, field delineation has several important psychosocial implications for our community. Currently, it can sometimes be unclear who is considered a ‘member’ of the medical education field. This raises issues around whose voices are being heard and whose voices are absent from our ongoing conversations. For example, does medical education have representation from non‐English speakers, women and trainees? Related to this idea of membership and representation, it may be difficult for researchers themselves to claim an identity in medical education, which can confer a sense of belonging and ownership for researchers.^7^

Field delineation is rarely straightforward. Indeed, there is no foolproof approach for all fields and often field borders can be quite fuzzy.^12^, ^13^, ^19^ A field's border can be especially fuzzy in cases of emerging, interdisciplinary or multidisciplinary fields. In such cases, field delineation can be fraught with additional complications.^11^ For example, in an interdisciplinary field, a given journal may contain articles that address subject matter that cannot be easily assigned to a single field. Furthermore, an article about a given topic, for instance, a study of physicians' social media use, could appear in a medical education journal, a communication journal, or even a general medicine journal. Adding to the complexity of field delineation, it is often the users or actors in a domain who ultimately determine the boundaries of a field, which can introduce additional challenges and biases.^19^

In medical education, we utilise multiple epistemologies and underlying philosophies lack a specific medical education vocabulary, make available our scholarship in a variety of formats (e.g., peer‐reviewed articles, books, blogs, podcasts) and often orient our research in a local educational context. All of these complicating factors make medical education a difficult field to delineate. Nonetheless, we believe it is time to begin making progress toward field delineation in medical education. To that end, in this manuscript, we follow the lead of researchers from nanoscience, a similarly multidisciplinary field, who explored potential field delineation approaches for their field comparing and contrasting the approaches in light of their field's unique characteristics.^11^ In particular, we describe three bibliometric approaches, two focused on the identification of journals and one focused on identifying relevant articles, and conclude with a proposed ‘starter set’ of medical education journals. In doing so, our primary aim is not to propose a definitive set of publications that define the field of medical education, but rather to introduce readers to a form of field delineation that we hope will prompt future collaborative work to further delineate the field using other field delineation approaches (e.g., consensus methods).

## 2 PROCESS

### 2.1 Information retrieval

The information retrieval approach is a popular method of field delineation. For this approach, researchers attempt to identify all of the relevant articles in the field by searching the literature (i.e., information retrieval) such that the retrieved articles would be considered as a representation of the field. We consider this akin to conducting a search as part of a comprehensive systematic review. This approach has been used in several fields (e.g., nanoscience and information science),^10^, ^12^, ^19^, ^20^ as well as in medical education.^3^ In 2010, Lee and colleagues searched PubMed using the medical subject heading (MeSH), ‘education, medical’ as the major focus of articles.

Over a decade later, we loosely replicated Lee et al.'s approach using a broader search approach.^3^ To begin, we conducted a PubMed search for the keywords ‘medical education’. This search would retrieve any citations with this term in its metadata (e.g., title, abstract, author details), including any articles indexed with the MeSH term ‘education, medical’ and its more specific terms related to undergraduate, graduate and continuing medical education. At this point, we considered that this corpus of citations, which contains over 200 000 articles published across hundreds of journals, represents the field of medical education. Notably, we could have chosen to search other databases or multiple databases (e.g., Web of Science, Scopus and Google Scholar), but we chose PubMed for our exploration because it is free, includes MeSH, and is considered the ‘premier biomedical database’.^21^

While 200 000 citations is a solid initial set of citations, upon closer inspection of the citations retrieved, some limitations were immediately revealed. For example, this search retrieves the article: ‘Albuminuria intensifies the link between urinary sodium excretion and central pulse pressure in the general population’.^22^ This article, which seems to be unrelated to the field of medical education, is retrieved because the author's institution is Miyagi University of *Education Medical* Centre. We were also concerned about missing relevant articles. For example, *Academic Medicine*, which is often considered a core journal in the field^3^, ^4^, ^6^, ^23^ has published over 12 842 articles since its inclusion in PubMed. However, our strategy only retrieved 5824 citations from *Academic Medicine* meaning that over 7000 citations appear to be missing, including the seemingly relevant article: Toward a more perfect match: Improving the residency application process.^24^ We speculate that these missing articles are an artefact of the primarily human indexing process in which indexers, who likely do not have a background in medical education, select a finite number of MeSH terms based on what they perceive to be the most important elements of an article.

After examining the citations, this approach could be optimised by constructing a more comprehensive search string, possibly in collaboration with a medical librarian, to systematically remove some of the irrelevant citations retrieved in the search (e.g., search titles and abstracts only). Researchers could also expand their search by adding additional relevant terms such as ‘medical student’ or ‘medical school’. Similar to the approach taken in a systematic review, the researchers would most likely need to iterate their search through multiple rounds, which can be a resource‐intensive approach.

### 2.2 Core journals

In a second approach, we identify a collection of ‘core medical education journals’ to define the field. For example, consider the approach undertaken in social work in which the author identified 25 main journals in their field based on Clarivate's Journal Citation Reports' (JCR) subject classification.^25^ The JCR classifies over 12 000 journals into subject categories, including the category ‘social work’. This approach has been considered the ‘best way’ to identify core sets of journals^26^ and could be used by a researcher attempting to identify investigators who publish in a particular field or to characterise a field's key research topics. However, turning to the JCR, there is no subject category for ‘medical education’ and thus no preset list of journals. There is a somewhat close fit with journals characterised in the category: ‘education, scientific, disciplines’. However, this also contains titles such as *Engineering Education* and *American Journal of Physics*. Based on the journals' scope note descriptions and a review of the titles of articles published in 2020, these two journals appear to be outside of the medical education field, such that using this approach would introduce a fair amount of irrelevant content. We also investigated a second resource, the Scimago Journal and Country Rank, which includes a seemingly close topic: ‘social sciences, education’. Similar to the JCR, there were many journals in the resulting list that were well outside the scope of medical education (e.g., *Child Development*).

Next, we considered the ‘Annotated Bibliography of Journals for Educational Scholarship’, which was collated by the Medical Education Scholarship Research and Evaluation Section (MESRE), a special interest group of the Association of American Medical Colleges.^27^ This list aims to provide researchers and scholars with a sense of the topics, types of manuscripts and the audience for journals in the broad domain of health professions education. The list includes 67 journals and features many of the titles that are commonly referenced in medical education bibliometric studies,^1^, ^2^, ^3^ which is an encouraging finding. However, it also includes titles that focus on education in general (e.g., *AERA Open)*, allied health disciplines (e.g., *Journal of Dental Education*) and journals that are predominantly clinical, but include some education research (e.g., *JAMA*). While this is an incredibly valuable resource for individuals wanting to identify a place to publish, for our purpose of field delineation, we feel it is too broad. For example, in 2020 *JAMA* published over 15 000 articles of which only 1886 articles are indexed in PubMed as related to medical education. Therefore, the addition of more than 13 000 seemingly irrelevant articles would introduce quite a bit of noise into the journal set. Additionally, the construction of this bibliography reflects the leanings and preferences of its creators. As an AAMC product and with all the list's authors based in North America, the list tends to lean heavily toward North American and European publications, with the exception of *Focus on Health Professions Education*, which is the official journal of the Australian and New Zealand Association for Health Professional Educators.

### 2.3 Journal co‐citation

**FIGURE 1:**
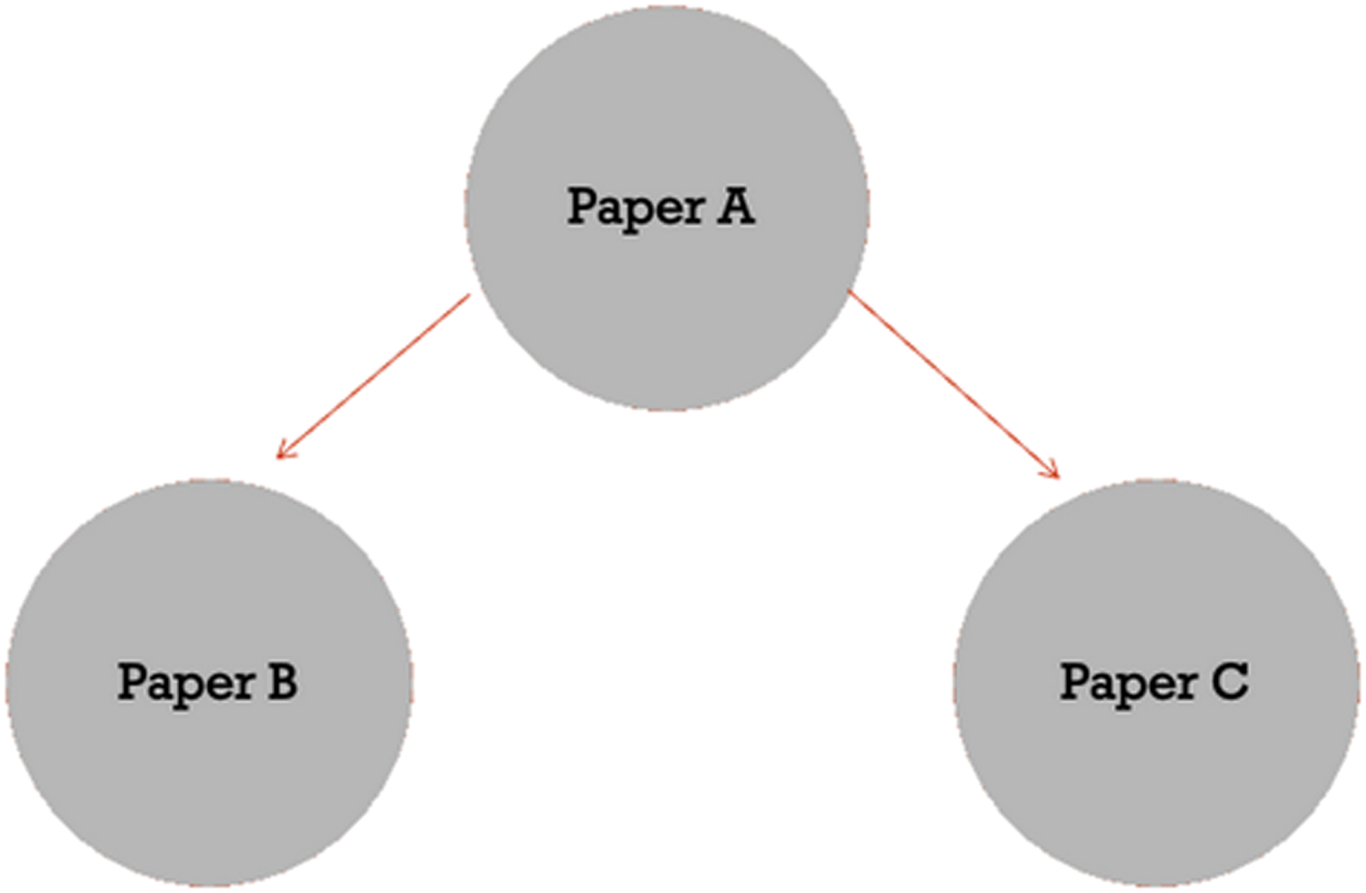
A basic example of co‐citation [Color figure can be viewed at wileyonlinelibrary.com]

In a third attempt, we utilised journal co‐citation. Journal co‐citation is the frequency with which two journals are both cited by a third journal. In this case, the two journals both cited by a third journal are considered to be ‘intellectually related’^28^ (See Figure 1). Co‐citation has been defined as a link between two entities (e.g., journals, journal articles and authors) by a third entity citing both.^28^ In other words, co‐citation is a measure of the ways in which authors use citations.^29^ For example, in Figure 1 we present an example of a basic case of a co‐citation relationship. In paper A, there are citations to both Paper B and Paper C. As a result of these two citations, we would refer to Paper B and Paper C as being ‘co‐cited’ by Paper A. This co‐citation serves as an indicator that these two papers are likely to be similar to one another. The more instances of Paper B and C being co‐cited by other papers (e.g., Papers D, E and F), the more likely they are to be similar.

To conduct co‐citation analysis requires a ‘seed set’ of journals and the metadata of their articles. Since such a set of medical education journals is currently unavailable, we decided to start with the 14 journals that have been described in the literature as ‘core medical education journals’.^1^, ^3^, ^23^ Using the JCR, we determined each journal's subject categories, which included ‘education, scientific and disciplines’ (*n* = 9); healthcare sciences and services (*n* = 6); education and education research (*n* = 2); and medicine, research and experimental (*n* = 1). It is important to note that we focused on the JCR subject classifications to enable an additional step of metadata extraction from Web of Science (WoS). We recognise that this choice introduces limitations that we will discuss later.

For each category, we downloaded the titles of each included journal, which resulted in 987 journals. We screened all journal titles for mentions of education, academia or teaching in the title. If the title was very generic (e.g., *JAMA*), we reviewed the journal's scope note to determine if ‘education’ was specifically mentioned. If education was mentioned in the note, the journal was included. This resulted in 24 journals (See supporting information Appendix A for journal list). However, at this point our approach hit a roadblock that required a trade‐off. In this set, two of the journals, *Journal of Graduate Medical Education* and the *Canadian Medical Education Journal*, are not indexed in WoS such that we were unable to retrieve the necessary metadata. Thus, these two journals were excluded from the seed set. Additionally, our approach identified the *Journal of General Internal Medicine* (*JGIM*) as being ‘in scope’. However, between 2000 and 2020, *JGIM* published 30 783 articles, of which only 2120 citations included ‘medical education’ when we search all fields. Thus, we made the decision to exclude *JGIM*, since only a minority of its articles (6.8%) focused on medical education. This left 22 journals.

We downloaded from WoS on 15 and 23 February 2021 the metadata for all articles published in these 22 journals between 2000 and 2020 (*n* = 34 768). Critical to the co‐citation approach, the metadata included the references to the articles that had cited the articles published in the 22 journals. To conduct the co‐citation analysis, we used VOSviewer.^29^ VOSviewer is an open source, freely available software that allows users to construct and visualise bibliometric networks based on co‐citation data. This tool has been used in multiple studies.^12^, ^30^, ^31^

Using VOSviewer, we identified that there were 66 833 instances of co‐citation in our set. Due to the large volume of data, VOSviewer prompted us to select a threshold for displaying co‐citations. Thus, we decided to focus on journals with articles that had been co‐cited at least 50 times. This resulted in 856 journals, which represented 318 591 citations. For a full listing of journal titles, see deposited data. By the frequency of co‐citations, the top three journals were *Academic Medicine* (*n* = 44 956), *Medical Education* (*n* = 24 434) and *Medical Teacher* (17475). These three journals accounted for over 25% of the co‐citations (See Table 1). The top 20 journals accounted for over 50% of the co‐citations, of which 9 journals were from the core set of 22 journals.

**TABLE 1:** Top 20 journals by number of co‐citations

Journal title	Dates indexed	No. articles in the seed set of journals	Co‐citations	Percent of total citations
*Academic Medicine* ^a^	1951 ‐	7816	44 956	14.11
*Medical Education*	1966 ‐	5529	24 434	7.67
*Medical Teacher*	1979 ‐	5071	17 475	5.49
*JAMA*	1945 ‐		10 439	3.28
*Anatomical Sciences Education*	2008 ‐	878	7277	2.28
*BMC Medical Education*	2008 ‐	3054	7105	2.23
*Journal of General Internal Medicine*	1986 ‐		6221	1.95
*The BMJ*	1922 ‐		6153	1.93
*Advances in Health Sciences Education*	1996 ‐	1196	5703	1.79
*The New England Journal of Medicine*	1928 ‐		5198	1.63
*Teaching and Learning in Medicine*	1996 ‐	1183	3625	1.14
*Annals of Internal Medicine*	1927 ‐		3539	1.11
*Clinical Anatomy*	1997 ‐		2948	.93
*The Journal of Continuing Education in the Health Professions*	2005 ‐	752	2828	.89
*Lancet*	1922 ‐		2693	.85
*The American Journal of Surgery*	1945 ‐		2636	.83
*Family Medicine*	2000 ‐		2596	.81
*Journal of Surgical Education*	2008 ‐	1705	2448	.77
*Journal of Graduate Medical Education*	Not Indexed		1959	.61
*Journal of Interprofessional Care*	2008 ‐		1829	.57
Total			162 936	51.14

*Note*: Total co‐citations journals = 66 833 with 318 591 citations based on the number of journals that were co‐cited at least 50 times.

^a^ Combined with citations from the *Journal of Medical Education*, which was *Academic Medicine's* previous title.

The 22 journals from the initial set accounted for 41.2% of co‐citations. Although due to database constraints noted above, we excluded two journals, *JGME* and the *Canadian Journal of Medical Education,* both were identified in the literature as ‘core journals’^3^, ^23^; these journals were co‐cited and we have included them in Table 2.

**TABLE 2:** Representation of the MEJ‐24, which comprises journals in the core set of 22 medical education journals plus the *Journal of Graduate Medical Education* and *Canadian Medical Education Journal*.

Rank	Journal^a^	No. articles in the seed set of journals	Co‐citations	Percent of Total
1	*Academic Medicine*	7816	44 956	14.11
2	*Medical Education*	5529	24 434	7.67
3	*Medical Teacher*	5071	17 475	5.49
5	*Anatomical Sciences Education*	878	7277	2.28
6	*BMC Medical Education*	3054	7105	2.23
9	*Advances in Health Sciences Education*	1196	5703	1.79
11	*Teaching and Learning in Medicine*	1183	3625	1.14
14	*Journal of Continuing Education in the Health Professions*	752	2828	.89
18	*Journal of Surgical Education*	1705	2448	.77
19	*Journal of Graduate Medical Education*	0	1959	.61
21	*Clinical Teacher*	1803	1819	.57
29	*Medical Education Online*	600	1343	.42
37	*GMS Journal for Medical Education*	394	1207	.38
45	*Simulation in Healthcare*	781	961	.30
48	*Advances in Medical Education and Practice*	981	928	.29
52	*Education for Health*	548	815	.26
60	*Perspectives on Medical Education*	612	686	.22
67	*International Journal of Medical Education*	485	629	.20
125	*Journal of Educational Evaluation for Health Professions*	372	346	.11
211	*African Journal of Health Professions Education*	392	199	.06
395	*Journal of Medical Education and Curricular Development*	272	105	.03
454	*Canadian Medical Education Journal*	0	91	.03
630	*Focus on Health Professional Education*	152	68	.02
677	*BMJ Simulation & Technology Enhanced Learning*	192	62	.02
	Total	34 768	133 290	41.84

^a^ This table does not include the *Journal of General Internal Medicine*.

Despite a lot of effort, this co‐citation approach also has limitations. First, the seed set of journals excluded journals not indexed in the JCR, including *JGME* and the *Canadian Medical Education Journal*. However, as both of these journals were both identified in the literature as core journals in medical education^3^, ^23^ and together accounted for .64% of the total co‐citations, we feel both of these publications warrant inclusion in the field of medical education. Additionally, due to indexing limitations, this approach does not take into account most specialty journals that focus on education, which tend to be indexed in relation to their specialty only. For example, *Academic Paediatrics* is indexed in only the category of Paediatrics despite the fact that the journal's scope note describes it as an ‘active forum for the presentation of pediatric educational research’.^32^

From a methods standpoint, a benefit of co‐citation is that it provides several ways of thinking about defining a field's core set of journals; however, this is also a limitation in that there is no gold standard approach to determining how to best interpret the results. In this article, we provided two interpretations, which did not produce what we would consider the ‘ideal set of journals’ to define the field of medical education. The first interpretation is based on the ‘top 20 journals’. This set includes nine journals from the core set but also introduces clinical journals (e.g., *JAMA* and *BMJ*). Although these clinical journals have some coverage of medical education, medical education research is a minority of the content covered in these journals. Thus, by including these clinical journals, we also introduce a good deal of irrelevant content.

The second interpretation was an attempt to determine how the original core set of journals performed. In other words, we tried to determine if these 22 journals greatly contributed to co‐citations. Because these journals plus *JGME* and the *Canadian Journal of Medical Education* contributed to 41.84% of the co‐citations, we propose that while not an ‘ideal set’ of journals, that they represent a starting point for delineating the field. We call this journal set the Medical Education Journals‐24 (MEJ‐24), based on the number of journals in the set.

### 2.4 Pearls

We propose that delineating medical education would provide valuable insights about the field in regards to conducting bibliometric studies, setting parameters for citation scores and for a researcher's ability to identify with the field. We make this proposal with the caveat that notwithstanding our best efforts, we agree with Munoz that there is no perfect means of field delineation^12^—at least not for a field like medical education with several complicating factors. In Table 3, we provide a listing of the pros and cons for each of the three approaches we attempted in this paper. Next, while we have tried to embed ‘practical pearls of wisdom’ throughout the manuscript, we focus on several key considerations for those considering similar projects using bibliometrics approaches and for those seeking to broadly delineate the field of medical education.

**TABLE 3:** Summary table of approaches described for delineating the field of medical education and the related pros and cons

Approach	Pros	Cons
Information Retrieval	Retrieves citations from across a range of journals Low‐ish effort From citation data a researcher could begin to characterise the field (author characteristics, publication types, topics, etc)	Retrieves papers that are outside of scope Misses relevant papers Generally requires searching multiple databases, some of which require a subscription
Definitive Journals	Retrieves citations only from the included journals A researcher could mine the journal set for publications that could be used to characterise the field If a definitive journal set exists this is low effort	Exclude articles not published in the journal set Contains the bias of those that created the journal set It is difficult to balance journals that include some medical education articles vs. those that focus on medical education
Co‐citation	Retrieves citations from across a range of journals Highly used and validated^26^ Offers a method to identify most important journals^26^	A high‐effort approach that may require consultation with a bibliometrician May require access to subscription databases Can miss journals that are related, but that do not cite the same resource Only works for articles with references and citations, which makes this less efficacious with newer articles that have not accrued citations

The three approaches described here have been used for field delineation for many years. However, recent advances in information and computer science have enabled researchers to expand these approaches. For example, researchers have used social network analysis and natural language processing to help make sense of the increasing amounts of available data.^13^ To this end, we encourage those interested in field delineation to explore emerging methods with the caveat that they strongly consider collaborating with researchers with expertise in information science, specifically those with expertise in bibliometrics.

In each approach, we necessarily relied on the available metadata for articles and journals. As we observed, this can be problematic for all three approaches. Therefore, it is important for researchers to examine the metadata prior to analysis to understand the strengths and weaknesses of their data set and to assess how this might impact their interpretations of the data. Furthermore, we would encourage journal editors to investigate how their journal is indexed. For example, should the editor of the *Journal of Academic Paediatrics*, which describes an education mission, seek to be indexed in WoS as an education journal in addition to its current indexing as only paediatrics? In addition to facilitating field delineation research, this may also facilitate the findability of the journal's content by those using educational search terms. Lastly, we acknowledge that we decided to focus on medical education for practical reasons (e.g., core journals have been previously identified in the literature^23^ and by a professional association^27^); however, it would be valuable for researchers to explore field delineation in relation to the broader field of health professions education.

While the use of bibliometric approaches for field delineation are valuable, it is important to bear in mind that these techniques are influenced by the research team's design decisions and interpretation of resulting data. As noted above, field delineation can be ‘fuzzy’ requiring that researchers make decisions that can vary between research teams, such as deciding if they will define a field in relation to specific journals or based on the content or topics of specific articles. For example, in the current work where we examined both journal‐ and article‐focused approaches, we ultimately propose defining medical education in relation to journals (i.e., the MEJ‐24). We felt that, although imperfect, a focus on journals that contain content collated by editors following education‐focused missions would be more on topic than relying on the indexing of those less familiar with the field. Therefore, we believe that an expanded research approach is needed, one that includes active discussions between a wide diversity of medical education stakeholders. We recommend that these discussions be structured with the aim of arriving at a working consensus on the scope of the field. To this end, we call on the community to use the MEJ‐24 as a starting point, or seed set of journals, to inform these critical conversations.

## FUNDING INFORMATION

No specific funding was received for this work.

## ETHICS STATEMENT

Reported as not applicable.

## DISCLOSURES

None reported.

## DISCLAIMER

The views expressed in this article are those of the authors and do not necessarily reflect the official policy or position of the Uniformed Services University of the Health Sciences, the Department of Defense or the US Government.

## Supporting information


**Data S1.** Supporting information

## Data Availability

None reported.
