# Supplementary material for: Delineating the field of medical education: Bibliometric research approach(es)
Source: Med Educ. 2021 Nov 2;56(4):387–94. doi: 10.1111/medu.14677 (PMC9298433; doi:10.1111/medu.14677)
Supplement: Supplementary file 1 — Data S1. Supporting information [file MEDU-56-387-s001.docx]

**Appendix A: Twenty-four journals identified**

| *Academic Medicine* |
| --- |
| *Advances In Health Sciences Education* |
| *Advances In Medical Education And Practice* |
| *African Journal Of Health Professions Education* |
| *Anatomical Sciences Education* |
| *BMC Medical Education* |
| *BMJ Simulation & Technology Enhanced Learning* |
| *Canadian Medical Education Journal* |
| *Clinical Teacher* |
| *Education For Health* |
| *Focus On Health Professional Education-A Multidisciplinary Journal* |
| *GMS Journal For Medical Education* |
| *International Journal Of Medical Education* |
| *Journal Of Continuing Education In The Health Professions* |
| *Journal Of Educational Evaluation For Health Professions* |
| *Journal Of Graduate Medical Education* |
| *Journal Of Medical Education And Curricular Development* |
| *Journal Of Surgical Education* |
| *Medical Education* |
| *Medical Education Online* |
| *Medical Teacher* |
| *Perspectives On Medical Education* |
| *Simulation In Healthcare-Journal Of The Society For Simulation In Healthcare* |
| *Teaching And Learning In Medicine* |
